# Molecular genetic study of triploidy and the hydatidiform mole in pregnancy loss: analysis of 10,000 consecutive cases

**DOI:** 10.18699/vjgb-25-67

**Published:** 2025-09

**Authors:** V.P. Pushkarev, A.S. Masycheva, E.A. Glazyrina, T.E. Serebrenikova, V.B. Chernykh

**Affiliations:** Medical Genetic Center LLC Progen, Moscow, RussiaMedical Genetic Center LLC Progen, Moscow, Russia; Medical Genetic Center LLC Progen, Moscow, Russia; Medical Genetic Center LLC Progen, Moscow, Russia; Medical Genetic Center LLC Progen, Moscow, Russia; Bochkov Research Centre for Medical Genetics, Moscow, Russia

**Keywords:** triploidy, hydatidiform mole (complete and partial), miscarriage, quantitative fluorescent PCR (QF-PCR), short tandem repeats (STR), триплоидия, пузырный занос (полный и частичный), невынашивание беременности, количественная флуоресцентная ПЦР (КФ-ПЦР), короткие тандемные повторы, short tandem repeats (STRs)

## Abstract

Approximately 10–15 % of clinically recognized pregnancies result in miscarriage, with chromosomal abnormalities identified in about 50 % of early pregnancy losses (PL). Triploidy accounts for approximately 12 % of all chromosomal abnormalities in miscarriages. The additional haploid set of chromosomes in triploidy may be of paternal (diandric triploidy) or maternal (digynic triploidy) origin. Diandric triploidy is associated with a partial hydatidiform mole (PHM), while pregnancies involving diploid embryos with two paternal genomes (and loss of the maternal nuclear genome) are the most common cause of a complete hydatidiform mole (CHM). The hydatidiform mole (HM) is the most prevalent form of gestational trophoblastic disease. Genotyping of products of conception (POC) is currently considered a reliable method for confirming HM and distinguishing its subtypes. The aim of this study was to use DNA genotyping of POCs to detect cases of triploidy, estimate the frequency of HM and its subtypes, and analyze the molecular and clinical characteristics of triploid pregnancies, CHM, and PHM in a Russian population. Between 2018 and 2024, a total of 10,000 consecutive PL cases were analyzed at the Medical Genetic Center Progen (Moscow). The main clinical indications included spontaneous miscarriage, missed miscarriage, and anembryonic pregnancy. DNA genotyping was performed using a five-color multiplex QF-PCR method, which included profiling of 26 autosomal STR markers, as well as DYS437, DXS6809, the SRY gene, and 30 markers from homologous regions located on different chromosomes. CHM was diagnosed based on the homozygosity of all STR markers. Triploidy was identified by analyzing peak area ratios of non-homozygous STR markers, which exhibited characteristic patterns of approximately 2:1 or 1:1:1. In our cohort, chromosomal abnormalities were identified in 58.8 % of all PL cases. Triploidy was detected in 8.3 % of the total sample, representing 14.3 % of all chromosomally abnormal POCs. Diandric triploidy accounted for 43 % of triploid cases. The prevalence of CHM was 0.11 %. The median age of women with triploidy was 32.1 years, and 27.9 years for those with CHM. Given the observed frequencies of PHM and CHM in our cohort, along with the relatively young maternal age associated with these conditions, enhancing current diagnostic protocols for HM – particularly through the incorporation of DNA genotyping of POCs – is essential for the effective prevention and timely diagnosis of post-molar malignant neoplasms in this population.

## Abstract (ru)

Из клинически признанных беременностей 10–15 % заканчиваются выкидышем, и около 50 % абортусов на ранних сроках беременности имеют хромосомные аномалии. Триплоидии составляют примерно 12 % от всех хромосомных аномалий абортусов. Дополнительный гаплоидный набор хромосом может быть отцовского (диандрическая триплоидия) или материнского происхождения (дигиническая триплоидия). Диандрическая триплоидия проявляется частичным пузырным заносом (ЧПЗ). Беременности диплоидными эмбрионами с двумя геномами отцовского происхождения (и потерей материнского ядерного генома) признаны наиболее частой причиной полного пузырного заноса (ППЗ). Пузырный занос (ПЗ) – это самый распространенный тип гестационной трофобластической болезни. Генотипирование абортусов в настоящее время рассматривается как надежный метод для подтверждения и дифференциальной диагностики подтипов ПЗ. Целью данного исследования было с помощью ДНК-генотипирования абортусов при невынашивании беременности (НБ) выявить случаи триплоидии, оценить частоту ПЗ, его подтипов, молекулярно- генетические и клинические особенности триплоидной беременности, ППЗ и ЧПЗ в российской популяции. С 2018 по 2024 г. в медико-генетическом центре «Проген» (Москва) были исследованы 10 000 последовательных случаев НБ. Основными направительными диагнозами являлись спонтанный выкидыш, неразвивающаяся беременность, анэмбриония. ДНК-генотипирование проводилось с помощью метода мультиплексной КФ- ПЦР, включавшего профилирование 26 аутосомных STR-маркеров, DYS437, DXS6809, SRY и 30 маркеров на гомологичных участках пар хромосом. Критерием ППЗ была гомозиготность всех STR-маркеров. Критерием триплоидии было соотношение площадей пиков всех негомозиготных STR-маркеров, близкое к 2:1 или 1:1:1. В нашей выборке из 10 000 случаев НБ аномальный кариотип абортусов был выявлен в 58.8 %, доля триплоидии составила 8.3 % от общего числа случаев или 14.3 % от абортусов с аномальным кариотипом. Доля диандрической триплоидии составила 43 %. Частота ППЗ была равна 0.11 %. Медианный возраст женщин с триплоидией был равен 32.1 года, с ППЗ – 27.9 года. Учитывая оцененную в нашей выборке частоту ЧПЗ и ППЗ и относительно молодой возраст женщин, у которых он встречался, необходимо совершенствовать имеющиеся методы диагностики ПЗ (включение ДНК-генотипирования) с целью адекватной профилактики и своевременной диагностики постпузырных злокачественных новообразований в данной возрастной группе.

## Introduction

Ten to fifteen percent of clinically recognized pregnancies end in miscarriage, with approximately 50 % of early pregnancy losses (PL) attributed to chromosomal abnormalities (Soler et al., 2017; Essers et al., 2023). Triploidy accounts for about 12 % of all chromosomal abnormalities identified in spontaneous abortions (Jenderny, 2014; Soler et al., 2017).

**Fig. 1.**
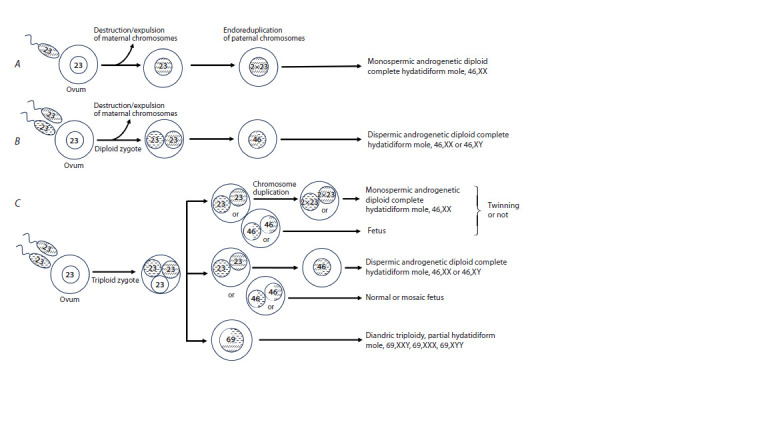
The main mechanisms of sporadic hydatidiform mole (HM) development. Complete hydatidiform mole develops after monospermic fertilization (85 % of cases) (A) or dispermic fertilization (15 % of cases) (B), where the maternal chromosomes were lost (or destroyed) immediately after conception. The result of the first scenario is an androgenetic diploid zygote with endoreplication of paternal chromosomes (A). C – post-zygotic diploidization of triploids (Golubovsky, 2003); a normal egg is fertilized by two sperm cells, resulting in a triploid zygote, which forms the basis for all types of HM and the fetus.

Triploidy is a genetic anomaly in embryonic or fetal cells characterized by the presence of three haploid sets of chromosomes (3n = 69) instead of the normal diploid number. The additional haploid set may be of paternal (diandric triploidy) or maternal (digynic triploidy) origin. The parental origin significantly influences the phenotypic manifestations of triploid pregnancies and maternal complications. Diandric triploidy most commonly arises from the fertilization of an ovum by two sperm cells (dispermy), or less frequently by a diploid sperm, and typically results in the development of a partial hydatidiform mole (PHM) (Fig. 1C). According to the concept of postzygotic diploidization of triploid cells proposed by M.D. Golubovsky in 2003, a normal ovum fertilized by two sperm cells may give rise to all types of hydatidiform mole (HM), as well as to a fetus. Sporadic complete hydatidiform mole (CHM) develops following monospermic (85 % of cases) or dispermic (15 %) fertilization of an ovum in which the maternal chromosomes are lost or destroyed shortly after fertilization (Fig. 1A, B). The result of monospermic fertilization is an androgenetic diploid zygote formed by endoreplication of the paternal genome (Candelier, 2016). In 10–20 % of cases, recurrent CHM is associated with biallelic pathogenic variants in maternal-effect genes. The list of implicated genes is steadily growing and currently includes NLRP7, KHDC3L, MEI1, TOP6BL, and REC114 (Murdoch et al., 2006; Parry et al., 2011; Nguyen et al., 2018).

The incidence of HM varies significantly across populations, ranging from 1–2 cases per 1,000 pregnancies in Europe and the USA to as high as 10 per 1,000 in India and Indonesia (Joyce et al., 2022). Both complete and partial HMs carry the potential for malignant transformation, with the risk of gestational trophoblastic neoplasia (GTN) being higher for CHM than for PHM (Joyce et al., 2022).In clinical practice, the main diagnostic tools for HM are elevated serum levels of β-human chorionic gonadotropin (β-hCG) – often tens of times higher than in normal pregnancies – and ultrasonographic findings. A definitive diagnosis is established through histopathological examination. However, these methods have limitations, particularly in early PL (Fukunaga et al., 2005; Sazhenova et al., 2009; Buza, Hui, 2021).

Genotyping of products of conception (POC) is currently considered a reliable approach for the confirmation and differential diagnosis of HM subtypes (Furtado et al., 2013; Ronnett, 2018; Buza, Hui, 2021). Distinguishing molar from non-molar specimens and differentiating PHM from CHM is critical for estimating the risk of post-molar GTN, which varies by HM subtype and determines the length and intensity of clinical follow-up (Buza, Hui, 2021).

The aim of the present study was to identify cases of triploidy using DNA genotyping of POCs from PL, to assess the prevalence of HM and its subtypes, and to characterize the molecular genetic and clinical features of triploid pregnancies, CHM, and PHM in the Russian population.

## Materials and methods

Between 2018 and 2024, a total of 10,000 consecutive cases of PL were analyzed in the laboratory of the Medical Genetics Center Progen (Moscow), with the majority of referrals originating from Moscow and the Moscow region. The primary clinical indications included spontaneous miscarriage, missed abortion, and anembryonic pregnancy. Informed consent was obtained from all patients.

Chorionic villi, fetal membranes, and fetal tissues were examined as biological material. DNA genotyping was performed using five-color multiplex quantitative fluorescent polymerase chain reaction (QF-PCR), which included the profiling of 26 autosomal STR markers (D1S1656, D2S441, D3S1358, D4S2366, D4S2408, D5S818, D6S1017, D6S474, D7S820, D8S1179, D8S1115, D9S2157, D10S1248, D10S1435, THO1, D12S391, D13S317, D14S608, D15S659, D16S539, D18S535, D19S253, D20S482, D20S1082, D21S1412, D22S1045), a Y-STR marker (DYS437), an X- STR marker (DXS6809), the SRY gene, and 30 additional markers targeting homologous regions of different chromosome pairs. The selection criteria for STR markers included an expected heterozygosity of ≥ 0.7 and no more than 12 alleles in the Russian population (Smolyanitsky et al., 2004; Pesik et al., 2014; Zavarin et al., 2019).

**Fig. 2.**
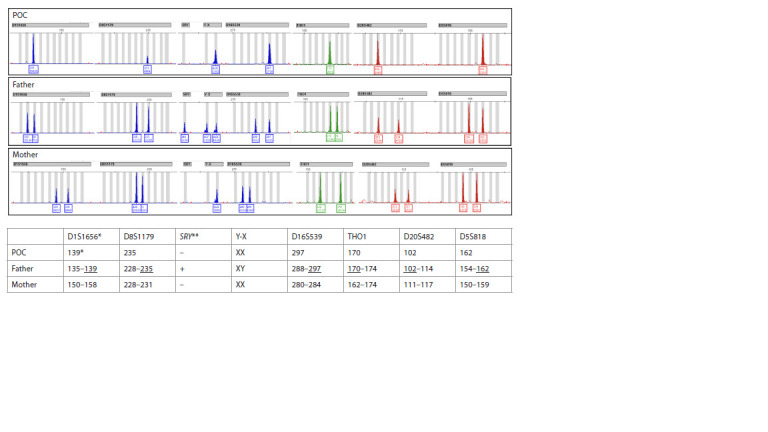
Identification of monospermic complete hydatidiform mole.

Note. * Length of alleles of informative STRs in nucleotides; ** Presence of SRY is indicated by a plus sign, absence by a minus sign. Shared alleles between the father's and POCs STR profiles are underlined. POC - product of conception.

**Fig. 3.**
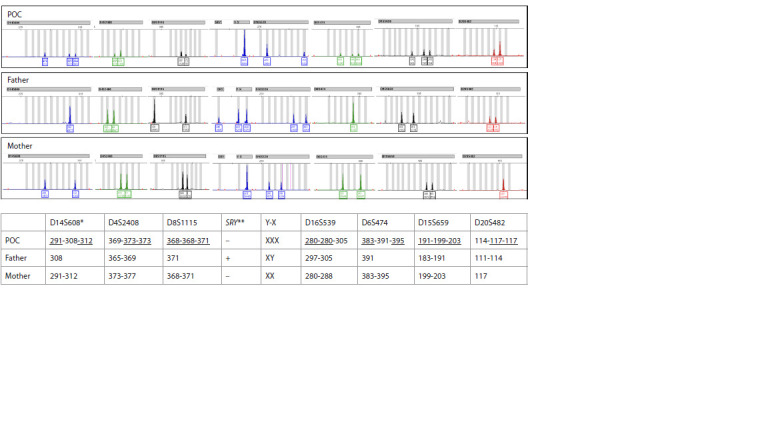
Identification of triploidy and determination of its origin (digynic triploidy). Note. * Length of alleles of informative STRs in nucleotides; ** Presence of SRY is indicated by a plus sign, absence by a minus sign. Shared alleles between the mother's and POC's STR profiles are underlined. POC - product of conception.

**Fig. 4.**
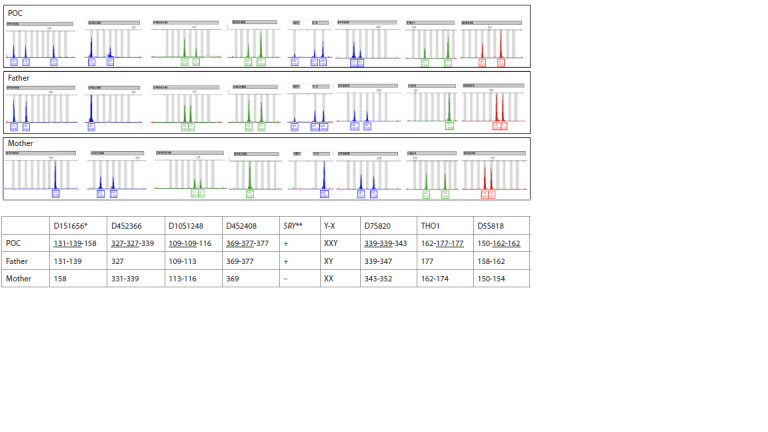
Identification of triploidy and determination of its origin (diandric triploidy).

PCR products were separated using a 3500 Genetic Analyzer (Thermo Fisher Scientific, USA), and electropherograms were analyzed with GeneMapper Software v5 (Thermo Fisher Scientific, USA). CHM was diagnosed based on homozygosity at all STR loci (Fig. 2), while triploidy was identified by peak area ratios of informative (heterozygous) STR markers approximating 2:1 or 1:1:1. The parental origin of triploidy (diandric or digynic) was determined by comparing the genotypes of the conceptus with those of the parents. The category "other chromosomal abnormalities" included autosomal monosomies Triploidy was identified in 829 cases (8.3 % of all PL cases; 95 % CI: 7.8–8.8). The median maternal age in this group was 32.1 years (IQR: 28.2–35.8), and the median gestational age at the time of PL was 8 weeks (IQR: 7–9). The parental origin of triploidy was determined in 14 cases: digynic triploidy in eight cases (57 %) and diandric triploidy (partial hydatidiform mole, PHM) in six cases (43 %). An example of digynic triploidy is presented in Figure 3, and that of diandric triploidy, in Figure 4. Our observed digynic-to-diandric ratio is consistent with previous reports; for example, D. Massalska et al. (2021) identified diandric triploidy in 44.9 % of triploid cases. and trisomies, sex chromosome aneuploidies, and complex karyotypic abnormalities. The "euploid karyotype" group included cases in which no chromosomal abnormalities were detected

Statistical analyses were performed using R software (version 4.4.2).

## Results

The median age of all women with PL was 34.6 years (interquartile range (IQR): 30.3–38.3 years). The median gestational age at PL was 7.5 embryonic weeks (IQR: 6.5–9). The summary of findings is presented in the Table.

**Table 1.**
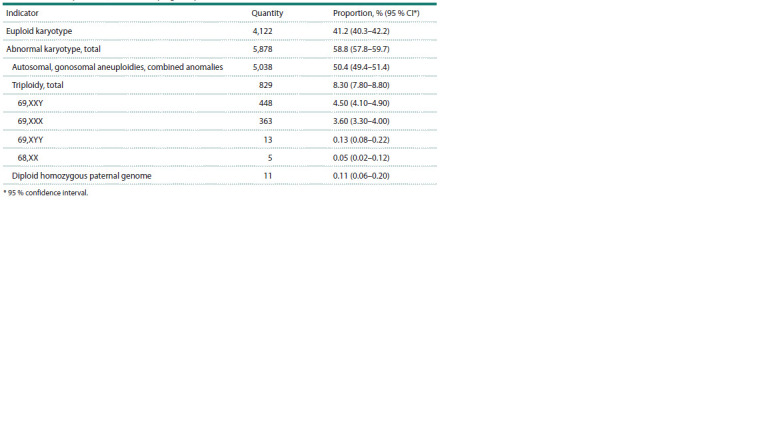
Results of the analysis of 10,000 cases of pregnancy loss * 95 % confidence interval.

Among 10,000 cases of PL, a normal (euploid) karyotype was identified in 4,122 samples (41.2 %; 95 % confidence interval (CI): 40.3–42.2). The median maternal age in this group was 33.5 years (IQR: 29.6–37.1), and the median gestational age at the time of PL was 7.5 weeks (IQR: 6.5–10).

An abnormal karyotype was detected in 5,878 cases (58.8 %; 95 % CI: 57.8–59.7). The median maternal age in this group was 35.4 years (IQR: 30.8–39.0), and the median gestational age at miscarriage was 7.5 weeks (IQR: 6.5–9). According to the Mann–Whitney U-test, there was a statistically significant difference in maternal age between the euploid and aneuploid groups (W = 8,233,198, p < 2.2 × 10–16).

Triploidy was identified in 829 cases (8.3 % of all PL cases; 95 % CI: 7.8–8.8). The median maternal age in this group was 32.1 years (IQR: 28.2–35.8), and the median gestational age at the time of PL was 8 weeks (IQR: 7–9). The parental origin of triploidy was determined in 14 cases: digynic triploidy in eight cases (57 %) and diandric triploidy (partial hydatidiform mole, PHM) in six cases (43 %). An example of digynic triploidy is presented in Figure 3, and that of diandric triploidy, in Figure 4. Our observed digynic-to-diandric ratio is consistent with previous reports; for example, D. Massalska et al. (2021) identified diandric triploidy in 44.9 % of triploid cases

**Fig. 5.**
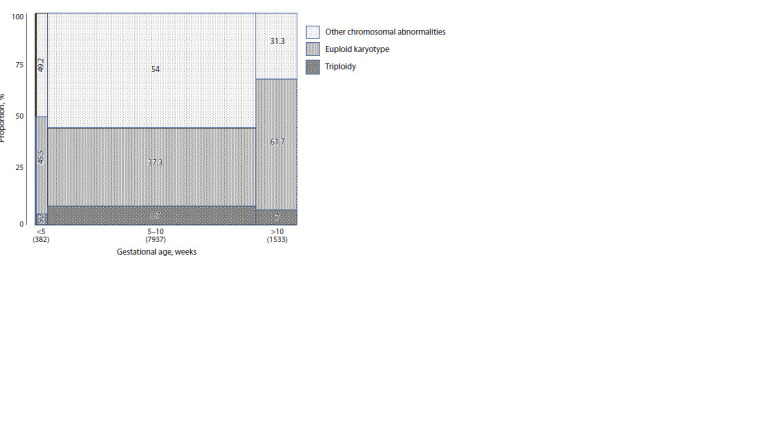
Distribution of study results by gestational age at pregnancy loss. The Y axis shows the proportion (%) and the X axis indicates gestational age at the time of pregnancy loss (in weeks). The total number of cases is shown in parentheses below each column. Gestational age was unavailable for 148 cases. The percentage labels on the bars indicate the proportion of each category among all cases at that specific gestational age. Bar width reflects the total number of cases at each gestational age. Other chromosomal abnormalities include autosomal and sex chromosome trisomies and monosomies, among others. Euploid karyotype refers to cases without chromosomal abnormalities. Triploidy indicates cases with a triploid karyotype

Complete hydatidiform mole was identified in 11 cases (0.11 % of all PL cases; 95 % CI: 0.06–0.20). Women in the CHM group were the youngest among all groups, with a median age of 27.9 years (IQR: 26.4–35.1). The median gestational age at the time of PL was 6.5 weeks (IQR: 6.5–7.5). In all 11 cases, homozygous STR profiles of the products of conception were observed, matching the paternal STR profile and differing from the maternal profile – consistent with a genome derived from a single sperm cell (Fig. 2).The majority of PL cases (80.6 %; 95 % CI: 79.8–81.3) occurred between gestational weeks 5 and 10 (Fig. 5). PL cases occurred before 5 weeks of gestation in 3.9 % of cases (95 % CI: 3.5–4.3), and those after 10 weeks occurred in 15.6 % (95 % CI: 14.9–16.3). The highest proportion of triploid cases occurred between 5 to 10 weeks (8.7 % of all PL cases). Autosomal and sex chromosomal aneuploidies, as well as combined numerical chromosomal abnormalities, were also most common in this time window – 54 % of all PLs. Euploid POCs were more frequently observed after 10 gestational weeks (61.7 %).

Statistical significance of the frequency distribution across gestational age groups was assessed using Fisher's exact test. The comparisons yielded the following p-values: "<5 weeks" vs. "5–10 weeks": p = 0.0012; "<5 weeks" vs. ">10 weeks": p = 8.6 × 10–10; "5–10 weeks"vs. ">10 weeks": p < 2.2 × 10–16.

## Discussion

In our cohort of 10,000 consecutive PL cases, molecular genetic analysis revealed chromosomal abnormalities in 58.8 % of samples, with triploidy accounting for 14.3 % of those with abnormal karyotypes. Similar proportions of chromosomal abnormalities and triploidy have been reported in studies analyzing chorionic villi from first-trimester miscarriages (Jenderny, 2014; Soler et al., 2017).

Approximately 80 % of PL cases occurred between gestational weeks 5 and 10 (Fig. 5). Classical clinical features of HM, such as vaginal bleeding and uterine enlargement, are rare at these early stages. This hampers the morphological differentiation between molar and non-molar tissue. It is estimated that 50 % of true PHM cases may be missed by routine histomorphology, with substantial inter- and intraobserver variability, even among experienced pathologists (Fukunaga et al., 2005; Hui et al., 2017). This may be due in part to the fact that trisomies involving chromosomes 7, 8, 13, 15, 16, 18, 21, and 22 can induce villous changes that mimic PHM (Buza, Hui, 2013; Gergely et al., 2024). These findings underscore the importance of DNA genotyping of POCs in differential diagnosis and in determining appropriate followup and prognosis for future pregnancies.

An increased incidence of CHM has been observed among women over 35 years of age and adolescent girls across different countries and ethnic groups (Hui et al., 2017). In our cohort, no bimodal distribution was observed; the CHM group (n = 11) had significantly younger maternal age than other groups. This could reflect sampling limitations or the low number of CHM cases detected.

Differentiating molar from non-molar pregnancies and distinguishing CHM from PHM is crucial for estimating the risk of post-molar gestational trophoblastic neoplasia, which varies by subtype and determines follow-up duration. CHM progresses to persistent/invasive mole in 15–20 % of cases and to choriocarcinoma in 2–3 %, while the risks for PHM are lower (0.5–5 % and 0.015 %, respectively) (Buza, Hui, 2021; Ul'rich et al., 2024). DNA genotyping of POCs is recognized as a reliable diagnostic method for HM, with validated clinical sensitivity and specificity for both CHM and PHM (Furtado et al., 2013; Buza, Hui, 2021).

We found limited data on HM frequency among PLs in the Russian Federation. An analysis of statistical data on early reproductive losses in the Ryazan region from 2017 to 2021 revealed that "hydatidiform mole was diagnosed in exceptional cases." (Aleshkina, Konovalov, 2023). In various autonomous districts of the Tyumen region, during the period from 2016 to 2021, HM accounted for 0.11–0.17 % of pregnancies with abortive outcomes before 12 weeks of gestation (Mateykovich et al., 2023). The total number of triploid cases identified by us (n = 829), as well as the proportion of diandric triploidy (43 %), allow us to estimate the number of PHM cases at approximately 350 per 10,000 cases of PL, or 3.5 %. These findings highlight the need to revise current diagnostic approaches. Early and accurate diagnosis of HM is crucial for reducing complications and preserving fertility in young women, given the risk of progression to persistent trophoblastic disease.

A limitation of this study is the inability to identify recurrent HM, which is an autosomal recessive condition.

## Conclusion

QF-PCR-based DNA genotyping of POCs reliably detects chromosomal abnormalities, including triploidy, CHM, and PHM. In our cohort of 10,000 PL cases, abnormal karyotypes were identified in 58.8 % of samples. Triploidy accounted for 8.3 % of all cases, or 14.3 % of those with abnormal karyotypes. The frequency of CHM was 0.11 %. The median maternal age in triploidy cases was 32.1 years (IQR: 28.2–35.8), while in CHM cases, it was 27.9 years (IQR: 26.4–35.1).

Given the observed frequency of both complete and partial HM in our cohort, as well as the relatively young age of the affected women, there is a pressing need to improve diagnostic protocols – particularly through the inclusion of DNA genotyping of POCs – to enable timely diagnosis and prevent post-molar malignant transformation in this age group.

## Conflict of interest

The authors declare no conflict of interest.
